# Effect of Plum‐Sour‐Based Marination Liquids Enriched With Linalool and Eugenol on the Microbiological and Sensory Quality of Chicken Breast Meat

**DOI:** 10.1002/fsn3.70361

**Published:** 2025-06-02

**Authors:** Merva Nur Atasoy, Bahar Tuba Findik, Hilal Yildiz

**Affiliations:** ^1^ Department of Food Engineering, Faculty of Engineering and Architecture Nevsehir Hacı Bektas Veli University Nevsehir Türkiye; ^2^ Department of Chemistry, Faculty of Arts and Sciences Nevsehir Hacı Bektas Veli University Nevsehir Türkiye

**Keywords:** active essential oil components, antimicrobial activity, chicken breast fillet, eugenol, linalool, marination

## Abstract

Herein, the effects of linalool and eugenol in a plum‐sour‐based marinade were investigated to extend the shelf life of vacuum‐packed chicken breast fillets by controlling pathogenic and spoilage microorganisms. Chicken breast fillets were marinated in a plum‐sour‐based marinade containing linalool or eugenol at a ratio of 0.15% or 0.30%, as determined by the results of in vitro antibacterial analysis. The pH and microbiological quality of the samples were assessed on 0, 3, 6, and 9 days of storage. The dominant microorganisms in the chicken breast fillets were found to be TVC and *Pseudomonas* spp. The addition of essential active components to the marinade resulted in significant antibacterial effects compared to the control sample on day 9 of storage (*p* < 0.001). The linalool‐added marinade was able to reduce the number of TVC and *Pseudomonas* spp. by 2.183 and 1.967 log units, respectively. On the other hand, the eugenol‐added marinade reduced TVC and *Pseudomonas* spp. counts by 1.893 and 2.097 log units, respectively. The number of LABs decreased by 0.97–1.68 log units in all test groups compared to the control on day 9 of storage (*p* < 0.001). Total coliform counts were below the detection limit (< 1 log_10_ CFU/g) in all experimental groups, while they increased in the control group during storage. The number of yeast and mold in the eugenol‐containing test groups reduced significantly in comparison to all tested groups. Sensory evaluation of the samples showed that the 0.15% eugenol marinade had higher scores than the linalool marinade for all parameters tested.

## Graphical abstract

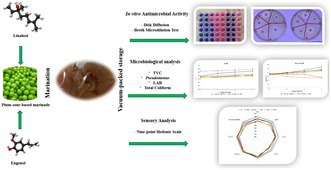

## 1 Introduction

Pathogenic microorganisms found in the natural environment of everyday life pose a threat to food safety, creating public health risks that lead to economic and social problems. With the increasing mobility of the population and the globalization of the food supply, food safety at every stage of food production, preparation, transportation, storage, and retailing has become a key issue around the world, requiring more precautions, especially for perishable products (Tsafrakidou et al. 2021). Poultry meat, one of the most important perishable products, has been in increasing demand worldwide over the past few years due to its low cost, high nutritional value, low fat content, and widely accepted taste (Cheng et al. 2023; Gurunathan et al. 2022). Despite its health benefits, poultry meat is susceptible to spoilage and deterioration due to its high protein and moisture content and acts as a reservoir for foodborne pathogens, especially *Salmonella* spp., *Campylobacter* spp., and *Listeria monocytogenes* . As a result, the quality and nutritional value of the chicken meat are compromised and even toxic or harmful substances are formed, which not only seriously affects the health of consumers, but also results in economic losses for producers and sellers (Souza et al. 2022).

Marination, a widely used traditional method in food processing, has found wide application in the meat industry. It serves as an efficient procedure that can create a safer microbiological environment, enhance the taste experience for the consumer, preserve the texture and flavor of the meat for a longer period of time, and reduce food waste, based on the specific formulation (Moon et al. 2017). Research on meat marinades has generally focused on thyme oil, lemon, vinegar, red wine, pomegranate juice, soy sauce, onion juice, grape juice, olive oil, pickles, and yogurt (Sengun et al. 2020). Moreover, the use of active essential oil components in marinating liquids has recently become a focus of interest to improve the flavor, aroma, and microbiological quality of the products.

Essential oils, which are naturally produced by plants as secondary metabolites, are characterized by their fragrant and volatile properties and offer a wide range of applications (Choi and Kim 2014). These oils include homologues of phenylpropanoids, coumarins, aldehydes, acids, hydrocarbons, alcohols, aliphatic lactones, and rarely nitrogen and sulfur compounds (Nazzaro et al. 2013). Of these components, secondary metabolites, such as monoterpenes and phenylpropanoids, have potential for use in the food industry (Silva et al. 2022). Phenylpropanoids, such as eugenol, are important components responsible for the aroma and flavor of foods (Abdollahi Mandoulakani et al. 2017). Monoterpenes, such as linalool, are associated with fruity, floral, minty, herbaceous, camphor, woody, pine, spicy, and citrus aromas that are thought to be critical to the flavor characteristics and acceptability of foods (Paulino et al. 2022). Monoterpene‐rich compounds or essential oils are also used in the agricultural, cosmetic, medical, and biotechnology industries. Despite extensive research in recent years on the in vitro antimicrobial activity of essential oils, the application of both essential oils and their active constituents in model food systems remains relatively unexplored. In a prior study conducted by our research group, it was found that the use of linalool and eugenol in marinating beef improved the microbiological quality, reduced lipid oxidation, and did not adversely affect the physicochemical properties of the meat (Bilen et al. 2024). On the other hand, only a handful of studies have investigated the effects of active essential oil components as marinating ingredients on the microbiological quality of chicken. A limited number of existing studies have examined the combined effects of active essential oil components, such as thymol and carvacrol, and packaging methods on the shelf life of marinated chicken (González‐González et al. 2021; Karam et al. 2019; Karatepe et al. 2024).

The plum ( *Prunus salicina* L.) belongs to the Rosaceae family and is known for its pleasant color, delicious flavor, and abundance of biologically active compounds (Sheikh et al. 2023). Plums are usually eaten as fresh fruit due to their perishable nature. To prevent quick spoilage, they are processed into juices, jams, or preserves (Soares Mateus et al. 2023). One of the common processing methods in Türkiye is plum sour, a homemade product that is obtained by concentrating the plum extract through heat treatment. Plum sour, with its aroma, flavor, and pH range, could be a promising candidate as a marinating liquid to meet consumer demand for “clean label” products.

As part of our ongoing efforts to use natural products/compounds to extend the shelf life of meat by improving its microbiological quality, we developed a new marination liquid by combining plum sour—which has a unique flavor and serves as a marinade base—with food‐grade bioactive components linalool and eugenol, aiming to enhance both the shelf life and sensory quality of poultry meat. The main problem encountered in the use of active essential oil components in foods is the undesirable organoleptic conditions caused by their strong flavor and aroma profiles. Therefore, preliminary in vitro antibacterial activity tests were conducted against eight different foodborne spoilage and pathogenic bacteria to determine the effective concentration of essential oils that could exhibit antibacterial activity without negatively affecting the sensory quality of chicken breast fillets. Taking into account the concentrations determined in the preliminary in vitro test, a marinating liquid was prepared by adding linalool and eugenol to the plum‐sour‐based marinade. After marinating chicken breast fillets for 24 h at 4°C, the marinated poultry meats were vacuum‐packed and stored under refrigerated conditions for 9 days, and the changes in microbial quality during the storage period were examined. The effect of the addition of linalool and eugenol on the organoleptic properties of chicken breast fillets was evaluated by sensory analysis.

## 2 Material and Methods

### 2.1 Material

The chicken breast fillets that were sold on the day of slaughter were obtained at a local butcher in Nevsehir, Turkey and transported in a cool box to the Food Microbiology Laboratory of Nevsehir Hacı Bektaş Veli University in Nevsehir, Türkiye. The active components of essential oils (linalool and eugenol, 97%) and Tween 20 were obtained from Sigma‐Aldrich (Darmstadt, Germany). The homemade plum sour used in the marination process was purchased from a local market in Samsun, Türkiye. All culture media were supplied by Merck (Darmstadt, Germany).

### 2.2 Determination of In Vitro Antibacterial Activities of Linalool and Eugenol

Agar disk diffusion and resazurin‐aided broth microdilution methods were used to determine the effective concentration of linalool and eugenol that showed antibacterial activity against eight reference strains (Food Engineering and Biochemistry Laboratories of Nevsehir Hacı Bektaş Veli University). For this aim, the strains that are prominent in meat spoilage and cause foodborne diseases were selected and are listed below:

Gram‐positive Reference Bacterial Strains: *Staphylococcus aureus* (ATCC 25923), *Listeria monocytogenes* (ATCC 7644) and *Bacillus cereus* (ATCC 11778).

Gram‐negative Reference Bacterial Strains: *Yersinia enterocolitica* (ATCC 27729), *Salmonella Typhimurium* (ATCC 14028), *Pseudomonas aeruginosa* (ATCC 35032), *Escherichia coli* (ATCC 25922), and *Escherichia coli* O157:H7 (ATCC 43897).

#### 2.2.1 Agar Disk Diffusion Method

The in vitro antibacterial activities of the active essential oil components were primarily detected using the agar disk diffusion method modified from the EUCAST Disk Diffusion Method for Antimicrobial Susceptibility Testing—Version 9.0, 2021. 10 μL of both linalool (97%, 858 μg/mL) and eugenol (97%, 1060 μg/mL) was applied to the disks (European Committe on Antimicrobial Susceptibility Testing (EUCAST) 2021). Because the active components of essential oils are volatile, the agar plates were covered with parafilm, kept at 4°C for 15 min to allow diffusion of the active component, and then incubated at 35°C for 18–24 h. After incubation, the diameters of the inhibition zones (DIZ) formed around the disks were measured in millimeters (mm) to determine their activity. Vancomycin (1 μg/μL) and ampicillin (1 μg/μL) were used as positive controls.

#### 2.2.2 Resazurin‐Aided Broth Microdilution Method

The minimum inhibitory concentrations (MIC: The lowest concentration of an antimicrobial reagent at which there is no visible growth of microorganisms) of active essential oil components were detected using the resazurin‐aided broth microdilution method adapted from the Clinical Laboratory Standard Institute guidelines (CLSI M07‐A9) (Wayne 2018). In a horizontal 96‐well U‐bottom microplate, columns 1–9 of the microplate were used for decreasing concentrations of the component to be tested, and columns 10–12 were used as controls (essential oil components, culture media, and growth control, respectively). Each sample was duplicated in a microplate, and the entire experiment was repeated three times with three different cultures of each bacterium.

The stock solutions of active essential oil components were prepared at a concentration of 0.8% (four‐fold the concentration to be tested; 8192 μg/mL) using 10% of dimethyl sulfoxide (DMSO) as a solvent and 0.5% of Tween 20 as an emulsifier. Therefore, the highest concentration of linalool or eugenol in the microplate was 0.2%. The first two rows (A1 and A2) of the microplate were used for the decreasing concentrations of DMSO and Tween 20 as a control. To monitor the inhibition of cell growth, 30 μL of 0.015% resazurin dye, a redox dye, was added to each well of the microplates and incubated at 35°C for 1 h. After incubation, a color change from blue to pink indicated the bacterial growth and the last blue well with no growth was determined as the MIC.

### 2.3 Investigation of the Effects of Linalool and Eugenol on Chicken Breast Fillets

Chicken breast fillets were used as a food model to determine the in situ efficacy of active essential oil components.

#### 2.3.1 Preparation of Marinade and Experimental Groups

A plum‐sour was used as the marination liquid which was prepared by homogeneously mixing plum‐sour and distilled water at a ratio of 1:5 (v/v). The mixture was divided into five parts to prepare experimental groups containing either linalool or eugenol at the ratios of 0.15% and 0.30%. These ratios were determined based on the MIC values obtained from the in vitro tests. The experimental groups are as follows:

C: Control (chicken breast fillets without any marinade application).

ML1: Marination liquid [plum‐sour and distilled water (1:5 ratio) without any essential active component].

ML2: ML1 + Linalool (L; v/v; 0.15%).

ML3: ML1 + Linalool (L; v/v; 0.30%).

ML4: ML1 + Eugenol (E; v/v; 0.15%).

ML5: ML1 + Eugenol (E; v/v; 0.30%).

Chicken breast fillets were cut into 15–20 g pieces on a sterile cutting board using a sterile stainless‐steel knife under aseptic conditions, and 400 g was used for each experimental group. For each experimental group, 400 g chicken breast fillets were marinated in the marination liquids at a ratio of 1:2 w/v in Ziplock plastic bags for 24 h at 4°C. At the end of the marinating process, the marinating liquids were removed from the samples. This day was accepted as the 0 day of storage. The control sample had the same storage conditions. Samples were vacuum packed in high‐density polyethylene bags and stored at 4°C for 9 days. Microbiological analyses were conducted at 0, 3, 6, and 9 days of storage.

#### 2.3.2 pH Analysis

The pH of the samples was measured using a digital pH meter (Thermo Scientific Orion 4‐Star Benchtop, Boston, USA). The pH meter was calibrated using standard solutions of pH 4.0, 7.0, and 10.0 (OrionTM pH Buffer Solution, Boston, USA). All measurements were performed at room temperature. The samples (10 g) were homogenized in deionized water (90 mL) using a laboratory blender (Waring Blender 8011ES). After equilibration for 10 min, the pH was measured.

#### 2.3.3 Microbiological Analysis

The samples (10 g), placed in a stomacher bag including 90 mL of sterile physiological peptone water (0.85% NaCl +0.1% peptone, w/v), were homogenized. After serial decimal dilution, the samples were spread (0.1 mL) or poured (1 mL) into the appropriate culture media to detect the total viable count (TVC) (PCA; spread plate method; 30°C, 72 h), lactic acid bacteria (LAB) (MRS agar containing cycloheximide‐0.1 g/L; the anaerobic conditions; spread plate method, 30°C, 72 h), *Pseudomonas* spp. (CFC agar with CFC selective supplement; spread plate method, 25°C, 48 h), total coliforms (VRB agar; pour double‐layer method; 35°C, 24 h), yeast and mold (PDA; spread plate method; 25°C, 5 days). The limit of detection was < 2 log_10_ CFU/g for the spread plate method and < 1 log_10_ CFU/g for the pour double overlay method because 0.1 mL and 1 mL dilutions were used, respectively. Each experiment was carried out in triplicate with three independent samples (Bilen et al. 2024).

#### 2.3.4 Descriptive Sensory Analysis of Chicken Breast Fillets

Chicken breast fillet samples were subjected to sensory evaluation by a panel of 15 individuals using a nine‐point hedonic scale based on aroma, color, flavor, taste, smell, tenderness, juiciness, and overall acceptability parameters. Fifteen panelists (8 females and 7 males) between the ages of 21 and 45 who were familiar with poultry consumption participated in the sensory test. The panel members were the students and staff of Nevsehir Hacı Bektas Veli University, who were trained about the procedures for conducting the sensory evaluations. A single‐blind approach was used for the sensory analysis. The samples were evenly cooked in a grill pan until the center of the piece reached 80°C and were immediately served to the panelists on a white plate, with random numbers assigned to the six samples. Water was served to the panelists for neutralization between samples. Analyses were performed in triplicate in different sessions. The grading scale ranged from 9 to 1 as follows: 9: Like extremely; 8: Like very much; 7: Like moderately; 6: Like slightly; 5: Neither like nor dislike: 4: Dislike slightly; 3: Dislike moderately; 2: Dislike very much; 1: Inedible.

### 2.4 Statistical Analysis

Statistical analysis was performed with SPSS 22 (IBM, SPSS Statistics, New York, USA). The data were subjected to analysis of variance (ANOVA). Differences between means were compared using Duncan's multiple comparison test.

## 3 Result and Discussion

### 3.1 In Vitro Antibacterial Activities of Linalool and Eugenol Against Foodborne Pathogenic Bacteria

The antibacterial activities of linalool and eugenol were evaluated by disk diffusion and resazurin‐aided broth microdilution tests.

#### 3.1.1 Disk Diffusion Analysis

The antibacterial activities of linalool and eugenol were found to be at various levels, and the DIZ values are presented in Table 1. According to CLSI‐M07A9 (2012), the breakpoints for susceptible, intermediate, and resistant isolates are set as ≥ 20 mm (susceptible: S), 19–15 mm (intermediate resistant: IR), and ≤ 14 mm zone of inhibition (resistant: R). Based on this standard, *Y. enterocolitica* (L: 85 mm, E: 40 mm), *E. coli* O157:H7 (L: 31.33 mm, E: 25.00 mm), *S. typhimurium* (L: 24.67 mm, E: 21.67 mm), *L. monocytogenes* (L: 25.67 mm, E: 23.00 mm), *B. cereus* (L: 26.67 mm, E: 20.33 mm) were found to be susceptible against both linalool and eugenol. Also, *E. coli* (21.33 mm) and *S. aureus* (22.33 mm) were determined to be susceptible to eugenol.

**TABLE 1:** Diameter of inhibition zone of the linalool and eugenol against foodborne pathogenic bacteria.

	Diameter of inhibition zone (mm)			
	Linalool (97%)	Eugenol (97%)	Vancomycin (1 μg/μL)	Ampicillin (1 μg/μL)
Gram (−) strains				
*Escherichia coli* O157:H7	31.33 ± 0.58^b^	25.00 ± 0.00^b^	10.67 ± 0.57^c^	00.00^c^
*Escherichia coli* (ATCC 25922)	14.67 ± 3.21^c^	21.33 ± 1.15^b^	19.67 ± 1.53^b^	23.33 ± 0.58^a^
*Pseudomonas aeruginosa* (ATCC 35032)	11.00 ± 0.00^c^	12.33 ± 3.79^c^	00.00^e^	00.00^c^
*Salmonella Typhimurium* (ATCC 14028)	24.67 ± 3.79^b^	21.67 ± 2.08^b^	8.00 ± 1.00^d^	12.00 ± 3.46^b^
*Yersinia enterocolitica* (ATCC 27729)	85.00 ± 0.00^a^	40.33 ± 0.58^a^	18.72 ± 1.15^b^	00.00^c^
Gram (+) strains				
*Bacillus cereus* (ATCC 11778)	26.67 ± 2.89^b^	20.33 ± 4.73^b^	22.67 ± 0.58^a^	11.00 ± 1.00^b^
*Listeria monocytogenes* (ATCC 7644)	25.67 ± 0.58^b^	23.00 ± 1.00^b^	22.00 ± 2.00^a^	11.33 ± 1.15^b^
*Staphylococcus aureus* (ATCC 25923)	16.33 ± 8.39^c^	22.33 ± 2.52^b^	22.68 ± 1.53^a^	24.67 ± 1.53^a^

*Note:* ^a–e^Within each column, values with different superscripts (lowercase letters) are significantly different (*p* < 0.000).

The findings demonstrated that *Y. enterocolitica* was the most susceptible of the foodborne pathogens to linalool and eugenol, with a statistically significant difference (*p* < 0.01) and *P. aeruginosa* (L: 11.00 mm, E: 12.33 mm) was found to be resistant to both linalool and eugenol.

Studies reported that both linalool and eugenol exhibited antibacterial activities at different levels against foodborne pathogens such as *Y. enterocolitica* (clove essential oil: 23.00 ± 3.00 mm), *E. coli* (L: 41.64 mm, E: 17.10 ± 1.20 mm), *S. aureus* (E: 12.70 ± 0.6 mm), *S. enterica* serovar Typhimurium DT104 (L: 18.60 mm), *S. typhimurium* ATCC 19430 (E: 20.10 ± 1.00 mm) (Goñi et al. 2009; González‐González et al. 2021; Guo et al. 2018; Zhang et al. 2017). The studies on the activity of linalool and eugenol against *P. aeruginosa* reported the resistance of *P. aeruginosa* to linalool and eugenol with the DIZ of 6–8.70 mm, respectively, which is consistent with our findings (Guo et al. 2018; Herman et al. 2016; Sanla‐Ead et al. 2012). No studies on the antimicrobial effect of eugenol on *Y. enterocolitica* have been found in the literature. The disparity between the results may be due to methodological differences, such as the use of different growth culture media, initial concentrations, and purity of the active ingredient (González‐González et al. 2021; Rivas et al. 2010).

#### 3.1.2 Resazurin‐Aided Broth Microdilution Assay

The MIC value of each active essential oil component was investigated by the resazurin‐aided broth microdilution method to evaluate the in vitro antibacterial efficiency, and the MIC values of linalool and eugenol against the tested microorganisms are presented in Table 2.

**TABLE 2:** The MIC values of the linalool and eugenol against foodborne pathogenic bacteria.

	MIC Value				
	Linalool (%)	Eugenol (%)	C1	C2 (μg/mL)	C3 (μg/mL)
Gram (−) strains					
*Escherichia coli* O157:H7	0.2	0.1	0.00	8.00	64.00
*Escherichia coli* (ATCC 25922)	> 0.2	0.2	0.00	0.50	4.00
*Pseudomonas aeruginosa* (ATCC 35032)	nd	nd	0.00	nd	256
*Salmonella Typhimurium* (ATCC 14028)	0.2	0.1	0.00	nd	16.00
*Yersinia enterocolitica* (ATCC 27729)	0.1	0.05	0.00	2.00	nd
Gram (+) strains					
*Bacillus cereus* (ATCC 11778)	0.1	0.1	0.00	0.25	128
*Listeria monocytogenes* (ATCC 7644)	0.1	0.1	0.00	> 0.50	32.00
*Staphylococcus aureus* (ATCC 25923)	nd	0.2	0.00	0.25	4.00

*Note:* C1: 10% DMSO +2% Tween 20; C2: Vancomycin; C3: Ampicillin; nd: Not determined.

Given that DMSO and Tween 20 were used as solvents and DMSO has an inhibitory effect on bacterial growth depending on the concentration, the initial two rows of microplate were designated as the solvent controls and no inhibitory activity was detected at the concentration used.

Eugenol showed the highest antibacterial activity against *Y. enterocolitica* with a MIC value of 0.05%. The results obtained against other strains showed a moderate level of activity, with MIC values ranging between 0.1% and 0.2%. Previous studies have also shown similar antibacterial activity of linalool and eugenol. MIC values of 0.0625% and 0.6% of linalool against *E. coli* O157:H7 were reported (González‐González et al. 2021; Shah et al. 2013). Zengin and Baysal (2014) reported a MIC value of 1000 μg/mL against two different *E. coli* O157:H7 strains (Zengin and Baysal 2014). In a study, the MIC of linalool at a concentration of 40 mg/mL prepared using 3% Tween‐20 as an emulsifier was reported to be 0.63 mg/mL against the *E. coli* ATCC 25922 (Varia et al. 2020). The MIC values of 0.7% and 1.25% were also reported for *S. typhimurium* (Prakash et al. 2019; Zengin and Baysal 2014). Indeed, variations in results among studies could be due to methodological differences, such as the use of different growth culture media and bacterial strains (Rivas et al. 2010). These variations can significantly impact the observed outcomes and should be considered when interpreting and comparing the results of different studies.

### 3.2 Effects of Linalool and Eugenol on Microbial Quality of Chicken Breast Fillets

Chicken breast fillets were chosen as a model food for the in situ assay of linalool and eugenol, the activities of which were determined in vitro. The samples were marinated and stored in a refrigerator for 9 days, and pH measurements and microbiological analyses (TVC, LAB, *Pseudomonas* spp., coliforms, yeast and mold) were performed on days 0, 3, 6, and 9 of storage.

#### 3.2.1 pH Analysis

**FIGURE 1:**
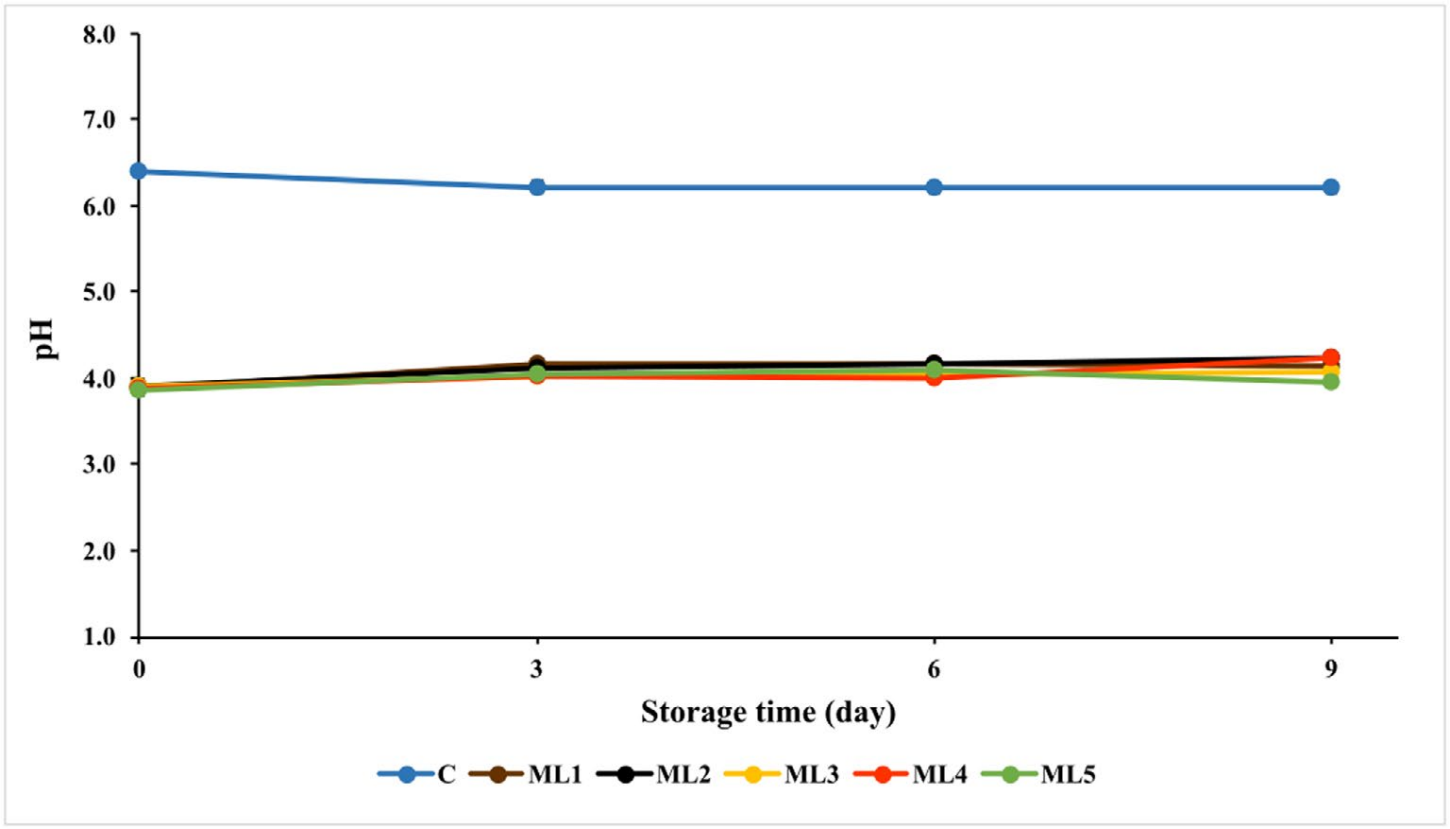
pH values of control and marinated chicken breast fillet samples during storage period at 4°C. C (Blue): Control (chicken breast fillet without marination); ML1 (Brown): Marination liquid [plum sour and distilled water (1:5 ratio)]; ML2 (Black): ML1 + linalol (L; v/v; 0.15%); ML3 (Yellow): ML1 + linalol (L; v/v; 0.30%); ML4 (Red): ML1 + eugenol (E; v/v; 0.15%); and ML5 (Green): ML1 + eugenol (E; v/v; 0.30%). Values are expressed as mean ± standard error.

pH is one of the most important parameters that directly affects the quality parameters of poultry meat products, such as tenderness, water‐holding capacity, color, juiciness, freshness, microbiological load, and shelf life (Rivas et al. 2010). Therefore, the degree of spoilage of meat can be evaluated by monitoring its pH (Mir et al. 2017). The changes in the mean pH values of the control and marinated chicken breast fillets during 9 days of vacuum‐packed storage at 4°C are shown in Figure 1 and Table S1.

The pH values after marinating for 24 h (0 day) of the control and experimental groups were recorded as 6.40 ± 0.03, 3.89 ± 0.02, 3.90 ± 0.07, 3.90 ± 0.03, 3.97 ± 0.02, and 3.85 ± 0.03 for C, ML1, ML2, ML3, ML4, and ML5 groups, respectively.

The pH value of the control sample varied between 6.20 ± 0.03–6.40 ± 0.03 during storage, and a statistically significant difference was found only on day 0 of storage (*p* < 0.001). The pH values of the marinated samples varied between 3.85 ± 0.03 and 4.23 ± 0.03 during storage. This difference in pH values between the control and marinated samples was due to the pH (2.89) of the marinating base (1:5 plum syrup: distilled water). The relatively small changes in pH observed in the experimental groups during storage are likely due to the buffering capacity of the meat (Björkroth 2005). Additionally, the decrease in LAB and the inhibition of coliform bacteria in the experimental groups during storage may have caused the increase in pH. As a matter of fact, the increase in the number of these microorganisms in the control group could be one of the reasons for the decrease in its pH, since these microorganisms produce acid by using carbohydrates.

Yildiz‐Turp and Serdaroglu (2010) observed similar decreases in the pH of low‐fat beef meatballs with the addition of plum puree at different ratios (5%, 10%, or 15%) (Yildiz‐Turp and Serdaroglu 2010). In another study, it was reported that the pH values of chicken breast meat samples marinated with different concentrations of apple and plum juices were lower than those of the control sample (Jarvis et al. 2015). The pH of the control sample was 5.89, while the pH of the marinated chicken breast meat was 3.69–3.71 in apple juice and 3.21–3.24 in plum juice (Erge et al. 2018). In the present study, the essential oil components added to the marinade did not significantly affect the pH of the sample. These results are similar to those of a study on the effects of thyme and garlic essential oils on the quality of chicken meat conducted by Kirkpinar et al. (2014), indicating no significant effect of essential oils on pH (Kirkpinar et al. 2014).

#### 3.2.2 Total Viable Count (TVC)

**FIGURE 2:**
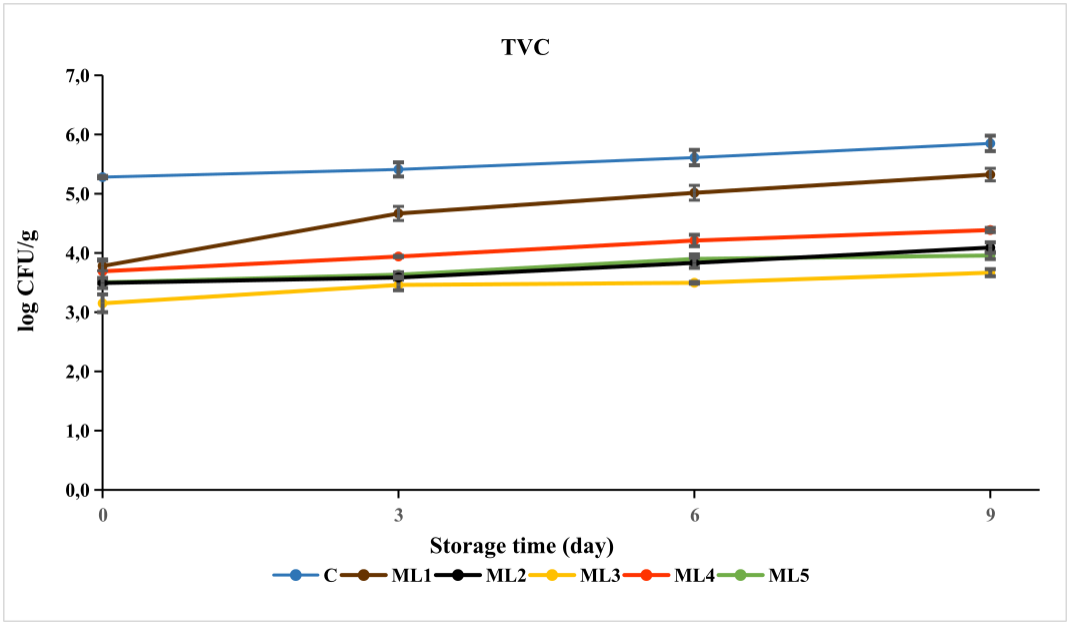
The growth of total viable count (TVC) in untreated and marinated chicken breast fillet samples during storage at 4°C. C (blue): Control (chicken breast fillet without marination); ML1 (brown): Marination liquid [plum sour and distilled water (1:5 ratio)]; ML2 (black): ML1 + linalol (L; v/v; 0.15%); ML3 (yellow): ML1 + linalol (L; v/v; 0.30%); ML4 (red): ML1 + eugenol (E; v/v; 0.15%); and ML5 (green): ML1 + eugenol (E; v/v; 0.30%). Values are expressed as mean ± standard error.

TVC are used as indicator microorganisms to determine the shelf life and sanitary quality of food, as their presence indicates food contamination (He and Sun 2015; Park and Kim 2013). The TVC counts of the control and experimental samples stored under vacuum at 4°C for 9 days are presented in Figure 2 and Table S2.

In the present study, the TVC count of the control was determined to be 5.28 ± 0.02 log CFU/g on day 0 of storage, which was below the acceptable upper limit of TVC count in raw poultry meat (≤ 6.48 log CFU/g) (Mahami et al. 2019). The TVC count of the control samples reached 5.85 ± 0.13 log CFU/g with a slight increase of 0.57 log CFU/g at the end of the 9‐day storage period, indicating the importance of the vacuum‐packed storage. According to Karam et al. (2019), vacuum packaging resulted in an almost 3 log CFU/g reduction in TVC counts of chicken meat stored at 4°C for 21 days compared to normal packaging (Karam et al. 2019).

After marinating for 24 h (day 0), the number of TVC in the marinated chicken fillets ranged from 3.15 to 3.78 log CFU/g. The reduction in TVC counts in all experimental groups compared to the control was due to the acidic nature of the marinade (pH 2.89 ± 0.02). The TVC count was found to be lower in all experimental groups compared to the control group at the end of storage (*p* < 0.01).

The plum sour‐based marinade (ML1) showed a decrease of 0.53 log units on the final day of storage compared to the control. The addition of active components of essential oils to the plum sour‐based marinade resulted in a significant reduction in the number of TVC on the final day of storage depending on the concentration. Linalool (ML2: 1.76 log units; ML3: 2.18 log units) was found to be more effective on the TVC counts compared to eugenol (ML4: 1.46 log units; ML5: 1.89 log units). The findings indicated that the antibacterial potential of essential oil components in situ applications was independent of the acidity factor, as the groups containing active essential oil components and marinade base had lower TVC counts than ML1 (marinade base) by an average of 1.00 log units on days 3, 6, and 9 of storage.

A study reported that the TAMB count in untreated chicken wings was 4.69 log CFU/sample on day 0 and reached 7.15 log CFU/sample after 7 days of storage, with an increase of 2.46 log units compared to the first day. On the other hand, the TAMB counts of chicken wings decreased during all storage days due to the addition of eugenol to the prepared coating materials. The results of this study showed that the number of microorganisms decreased with increasing concentration of eugenol (López‐Romero et al. 2018).

#### 3.2.3 *Pseudomonas* spp. Count

**FIGURE 3:**
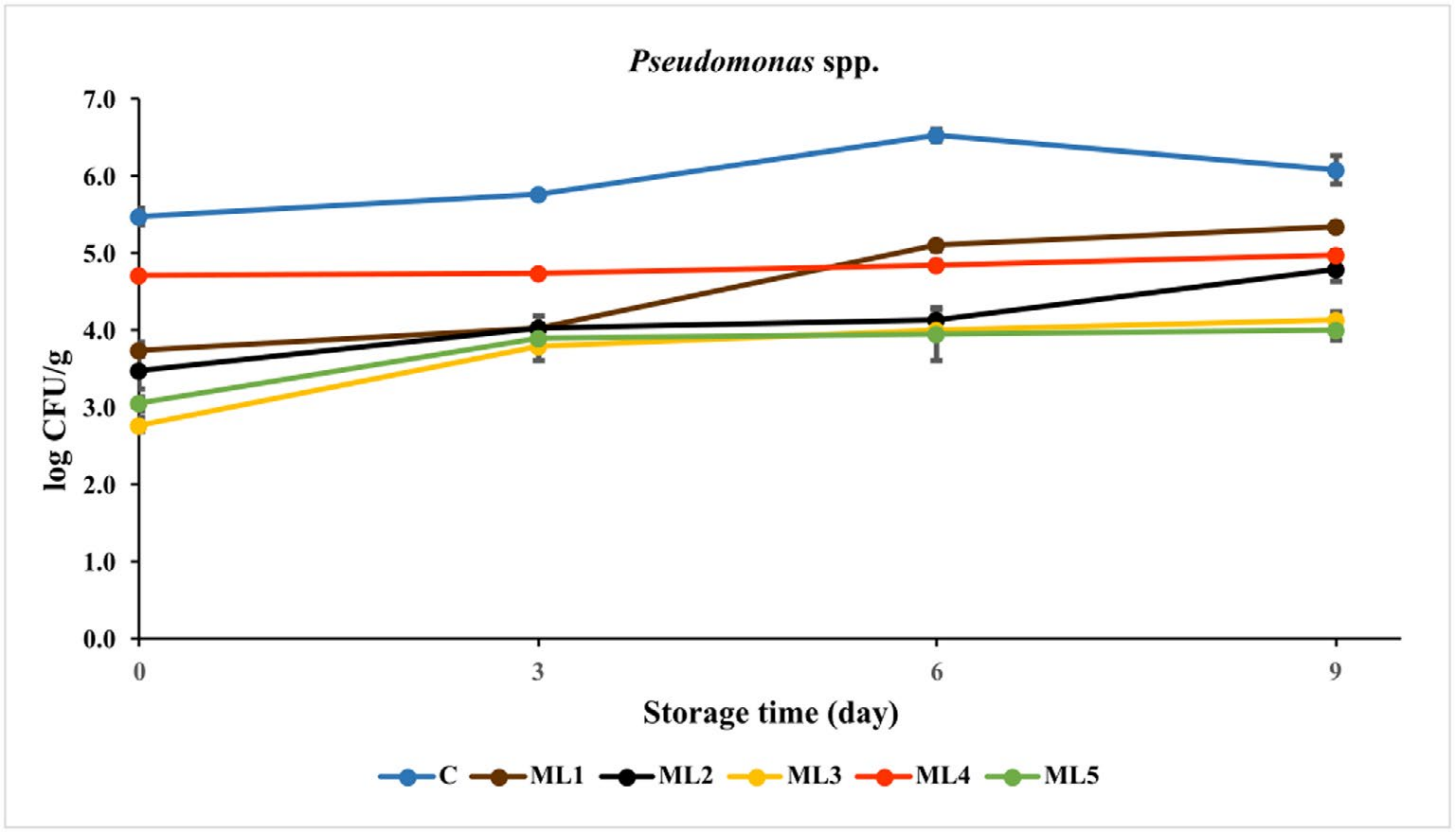
The growth of *Pseudomonas* spp. in untreated and marinated chicken breast fillet samples during storage at 4°C. C (blue): Control (chicken breast fillet without marination); ML1 (brown): Marination liquid [plum sour and distilled water (1:5 ratio)]; ML2 (black): ML1 + linalol (L; *v/v*; 0.15%); ML3 (yellow): ML1 + linalol (L; *v/v*; 0.30%); ML4 (red): ML1 + eugenol (E; *v/v*; 0.15%); and ML5 (green): ML1 + eugenol (E; *v/v*; 0.30%). Values are expressed as mean ± standard error.

*Pseudomonas* species are aerobic/facultative anaerobic bacteria that are important spoilage microorganisms in stored chilled foods due to their good adaptability and high protease and lipase activities. Since the growth of *Pseudomonas* spp. causes stickiness and unpleasant odor, leading to undesirable organoleptic changes and meat spoilage, the inhibition of *Pseudomonas* in foods is of particular importance (Marcelli et al. 2024; Radovanovic et al. 2020). The *Pseudomonas* counts of each group are given in Figure 3 and Table S3.

The initial *Pseudomonas* count of the control (day 0) was 5.47 ± 0.09 log CFU/g, and reached a value of 6.09 ± 0.18 log CFU/g on the last day of storage, below the upper microbiological limit for the quality of chicken meat (≤ log 7 CFU/g) (Monitoring and Surveillance Series 2010). All five treatments of chicken breast fillet were found to be effective against *Pseudomonas* spp. and showed significant reductions (*p* < 0.001) compared to the control during the storage period. The active components of essential oils at 0.3% (v/v) had the highest antibacterial activity in inhibiting *Pseudomonas* in chicken meat with a reduction of 1.97–2.10 log CFU/g on the 9th day of vacuum storage at 4°C.

As stated previously, the experimental groups in which linalool (ML2: 4.78 log CFU/g and ML3: 4.12 log CFU/g) and eugenol (ML4: 4.97 log CFU/g and ML5: 3.99 log CFU/g) added to the marination liquid showed better inhibitory activity on *Pseudomonas* spp. than the marinade base alone (ML1: 5.34 log CFU/g), demonstrating the effect of active components in addition to pH.

#### 3.2.4 Lactic Acid Bacteria (LAB) Count

**FIGURE 4:**
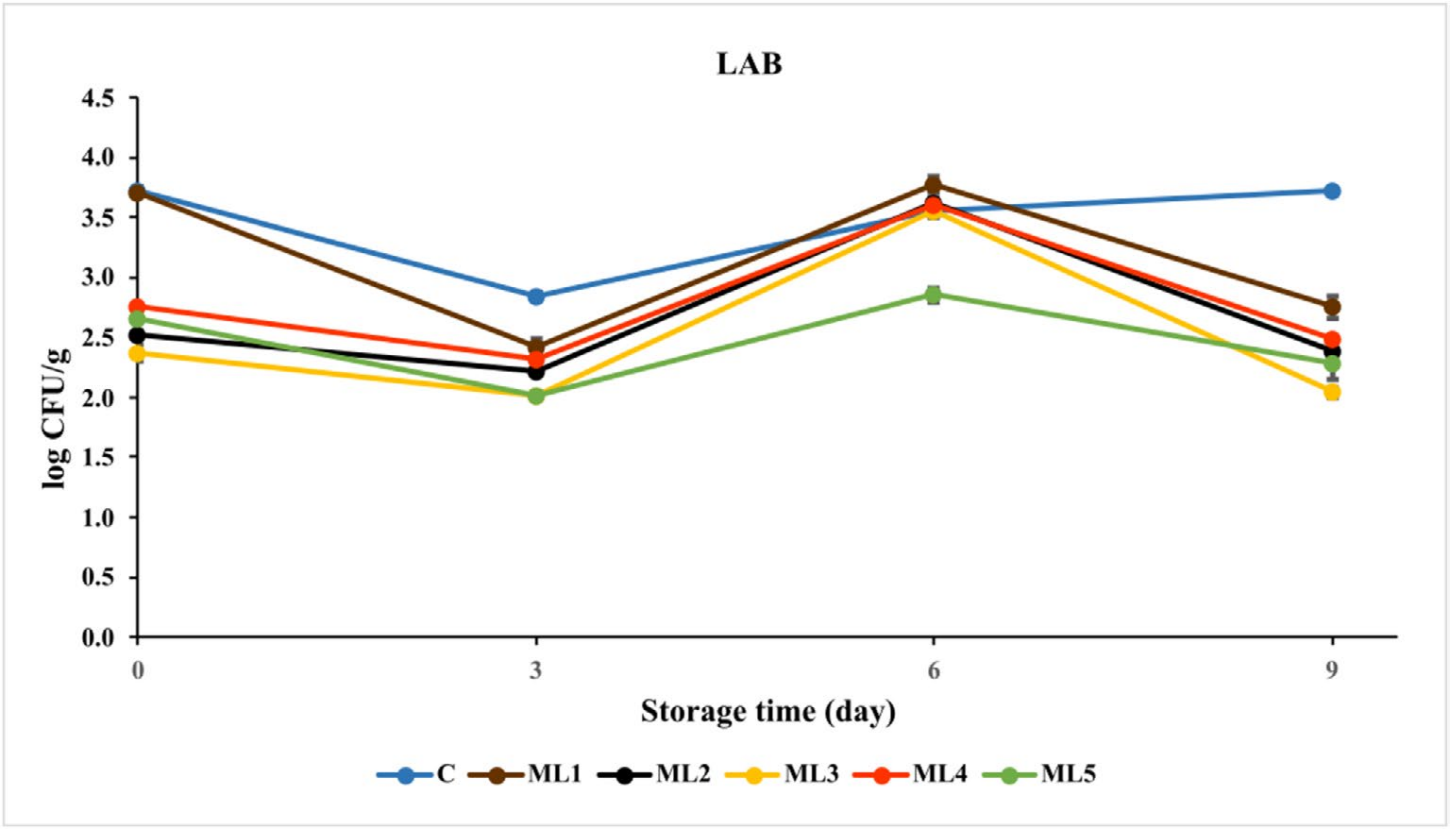
The growth of lactic acid bacteria (LAB) in untreated and marinated chicken breast fillet samples during storage at 4°C. C (Blue): Control (chicken breast fillet without marination); ML1 (Brown): Marination liquid [plum sour and distilled water (1:5 ratio)]; ML2 (Black): ML1 + linalol (L; v/v; 0.15%); ML3 (Yellow): ML1 + linalol (L; v/v; 0.30%); ML4 (Red): ML1 + eugenol (E; v/v; 0.15%); and ML5 (Green): ML1 + eugenol (E; v/v; 0.30%). Values are expressed as mean ± standard error.

LAB act as spoilage microorganisms in the meat microbiota during aerobic or vacuum storage of fresh meat (Fernández‐Pan et al. 2014; Karam et al. 2020). The LAB counts of each group are given in Figure 4 and Table S4.

Initially, the highest LAB count was found both in the control (3.70 ± 0.03 log CFU/g) and ML1 (3.69 ± 0.02 log CFU/g). All the experimental groups containing active components of essential oil had statistically significant (*p* < 0.001) lower LAB counts, and the differences were in the range of 0.96–1.35 log CFU/g. On the last day of storage, a statistically significant (*p* < 0.001) decrease in LAB count was observed in all groups except the control. On the 9th day of storage, the LAB count of the control sample was 3.72 ± 0.02 log CFU/g, while the lowest LAB count was found in chicken breast marinated with linalool (2.04 ± 0.04 log CFU/g) added to plum sour‐based marinade at a concentration of 0.30%.

In a study, chicken meat samples treated with thymol and carvacrol were stored for 21 days at 4°C under air and vacuum packaging. This study observed that the lowest LAB count was observed in the vacuum‐packed samples marinated with 0.8% carvacrol and thymol at the end of 21 days of storage. From day 6 of storage onwards, the number of LAB in the vacuum‐packed marinated chicken samples was found to be higher than that in the air‐packed samples (Karam et al. 2019). Considering these results, it can be said that vacuum packaging did not have a limiting effect on the growth of LAB due to its facultative anaerobic nature; however, the combination of active essential oil components limited the growth of this group of bacteria.

#### 3.2.5 Coliform Count

**FIGURE 5:**
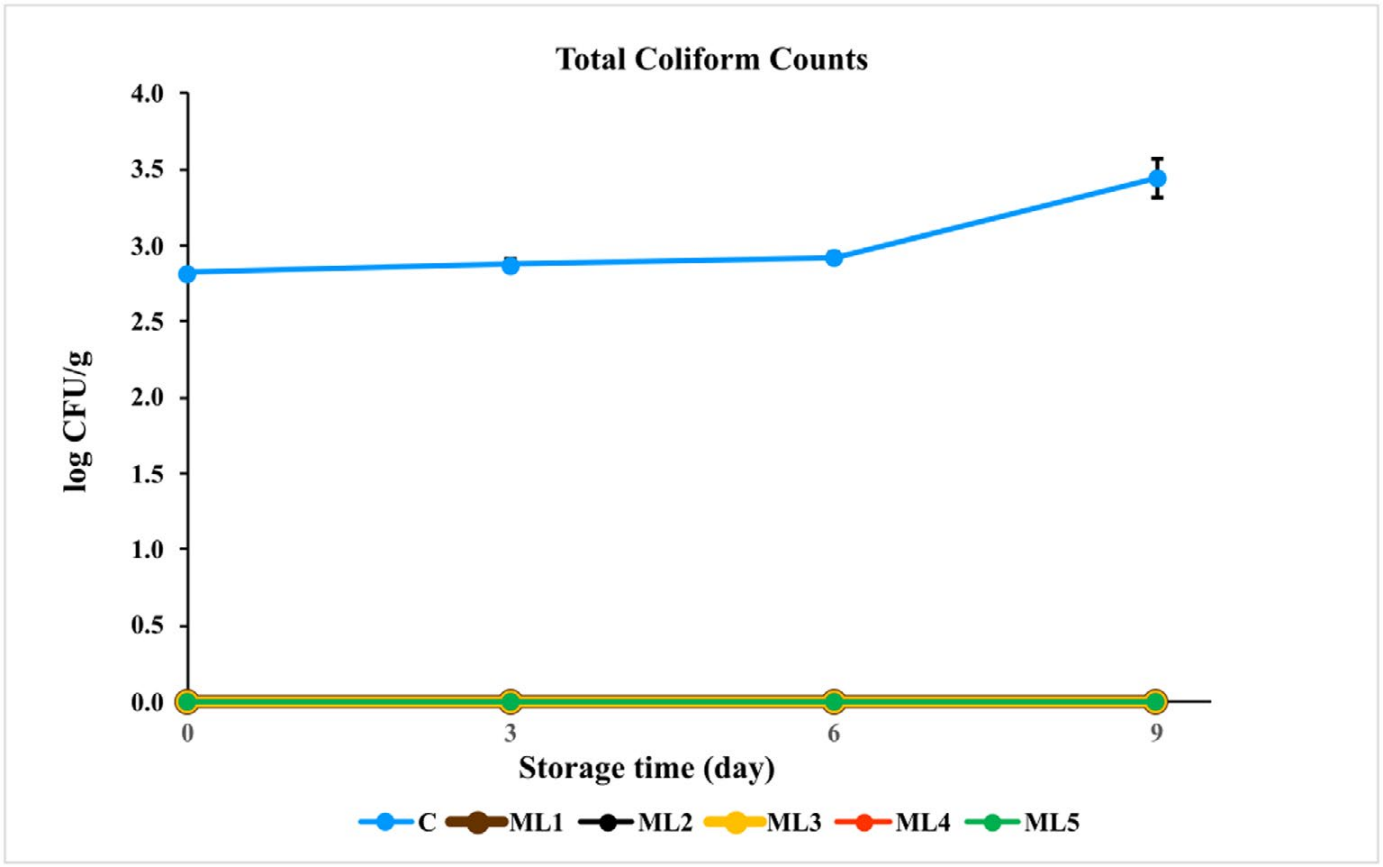
The growth of total coliform in untreated and marinated chicken breast fillet samples during storage at 4°C. C (blue): Control (chicken breast fillet without marination); ML1 (brown): Marination liquid [plum sour and distilled water (1:5 ratio)]; ML2 (black): ML1 + linalol (L; v/v; 0.15%); ML3 (yellow): ML1 + linalol (L; v/v; 0.30%); ML4 (red): ML1 + eugenol (E; v/v; 0.15%); and ML5 (green): ML1 + eugenol (E; v/v; 0.30%). Values are expressed as mean ± standard error.

Some microorganisms have been identified as potential indicators of hygienic food handling. In particular, the presence of facultative anaerobic coliform bacteria is a good criterion for determining the hygienic quality of meat. The coliform counts of each group are shown in Figure 5 and Table S5. In the study, total coliform counts during the storage ranged from 2.82 ± 0.03 to 3.45 ± 0.13 in the control group. Coliform counts were below the detection limit (< 1) in all experimental groups. These results are mainly due to the acidic nature of the marinade.

#### 3.2.6 Yeast and Mold Counts

**FIGURE 6:**
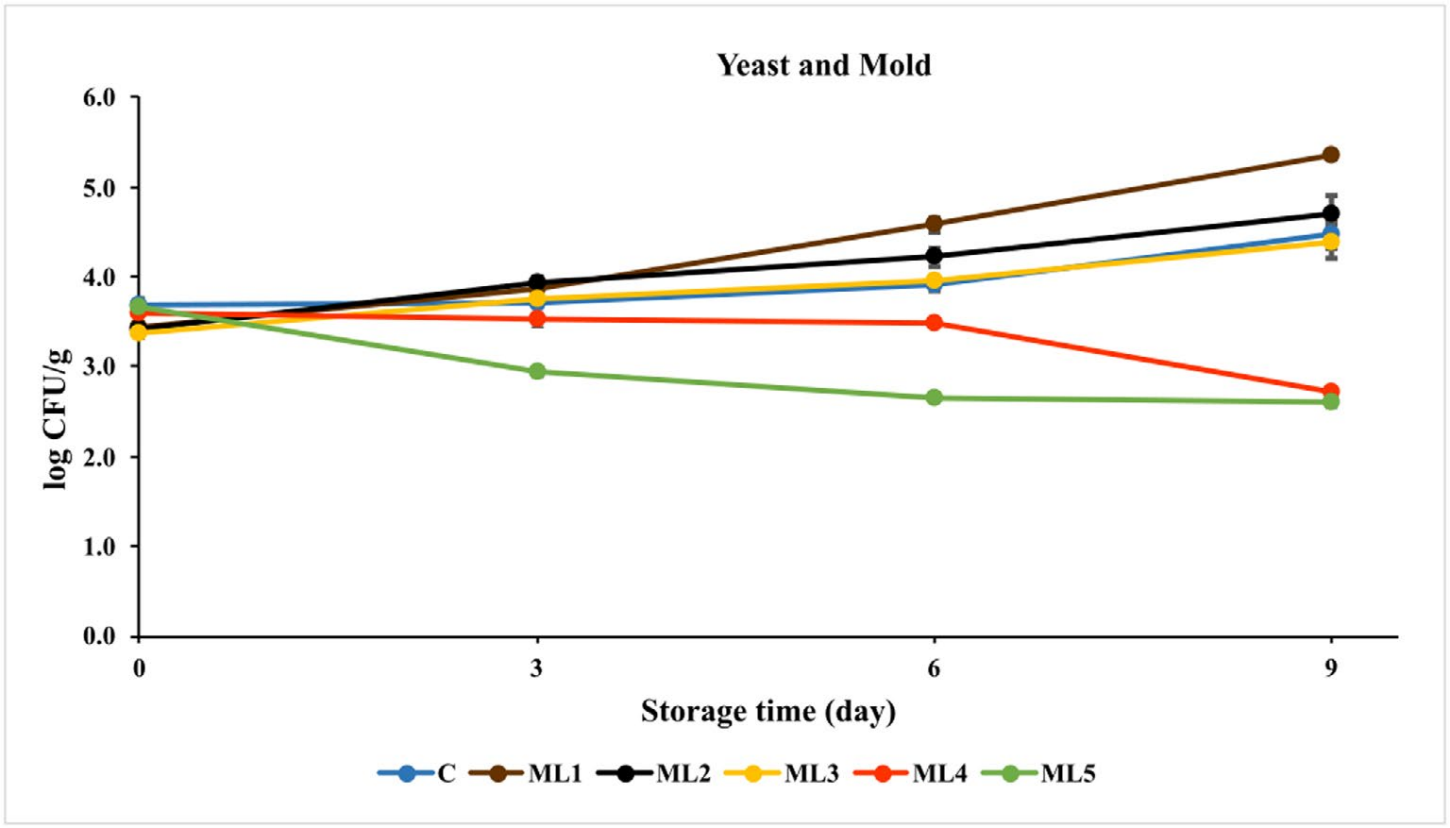
The growth of mold and yeast in untreated and marinated chicken breast fillet samples during storage at 4°C. C (blue): Control (chicken breast fillet without marination); ML1 (brown): Marination liquid [plum sour and distilled water (1:5 ratio)]; ML2 (black): ML1 + linalol (L; *v/v*; 0.15%); ML3 (yellow): ML1 + linalol (L; *v/v*; 0.30%); ML4 (red): ML1 + eugenol (E; *v/v*; 0.15%); and ML5 (green): ML1 + eugenol (E; *v/v*; 0.30%). Values are expressed as mean ± standard error.

The yeast and mold count for each group are shown in Figure 6 and Table S6. Mold was not found on all storage days, and the results given are for yeast.

At the beginning of the storage period, the highest yeast count was detected in the control group (3.69 ± 0.07). The yeast count of the other groups increased during storage except for the eugenol‐added groups (ML4 and ML5), and this increase was found to be statistically significant (*p* < 0.000). The addition of eugenol showed a statistically significant (*p* < 0.001) decrease in yeast inhibition. The use of eugenol at a ratio of 0.3% (ML5) showed a significant anti‐yeast effect, causing a final reduction of 1.04 log CFU/g compared to the initial day of ML5 and 1.87 log CFU/g compared to the final day of control.

In a study conducted on the use of different essential oils on vacuum‐packed fresh chicken sausages under frozen conditions as bio preservatives, it was reported that the number of yeasts and molds was not detected at the beginning of the frozen storage period and increased at the end of the storage period (45 days), except in the experimental group in which cassia oil was used (Sharma et al. 2017). In another study investigating the effect of a combination of carvacrol and thymol and different packaging models (air and vacuum) on the microbial flora of marinated meat, the number of yeast and mold was found to be approximately 3.00 log CFU/g or less depending on the concentrations of the active essential oil components used in the marinade. The results showed that the use of 0.8% carvacrol and thymol with vacuum packaging produced the best effect. However, there was still an increase in the number of yeast and mold at the end of storage compared to the first day of storage (Karam et al. 2019). When we compared our results with these studies, eugenol was found to be more effective in inhibiting yeast and mold as the number of yeast and mold decreased during storage. There are not enough data in the literature on the mechanism of action of active essential oil components against yeast‐mold, and it has been reported that the effect may occur as a result of interaction with the cell membrane, disruption of ergosterol biosynthesis, and cell homeostasis (Ca^2+^ loss) (Karam et al. 2020).

### 3.3 Descriptive Sensory Analysis of Chicken Breast Fillets

**FIGURE 7:**
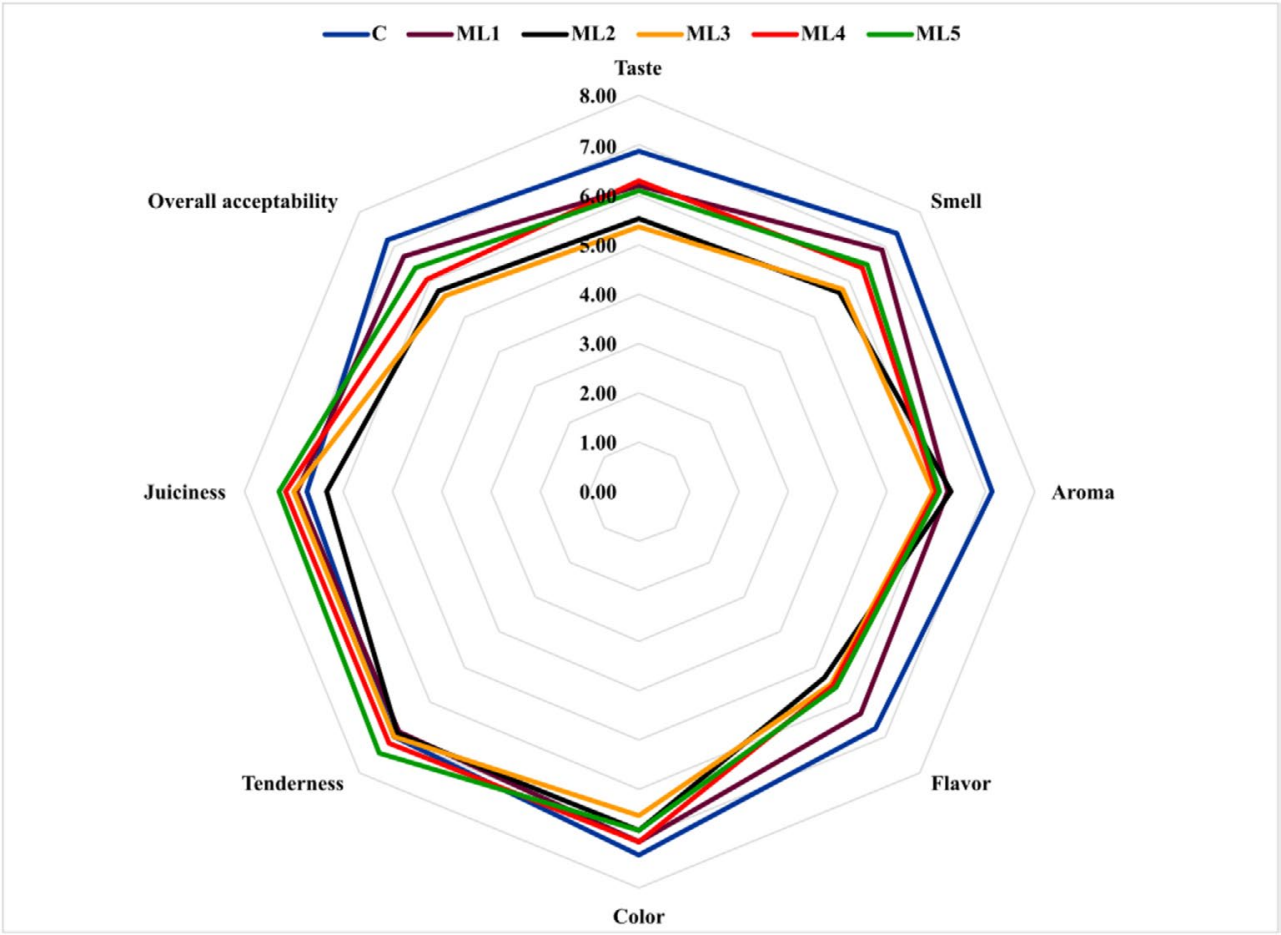
Radar plot of hedonic sensory evaluation of untreated and marinated chicken breast fillet samples. C (blue): Control (chicken breast fillet without marination); ML1 (brown): Marination liquid [plum sour and distilled water (1:5 ratio)]; ML2 (black): ML1 + linalol (L; v/v; 0.15%); ML3 (yellow): ML1 + linalol (L; v/v; 0.30%); ML4 (red): ML1 + eugenol (E; v/v; 0.15%); and ML5 (green): ML1 + eugenol (E; v/v; 0.30%). Values are expressed as mean ± standard error.

Sensory evaluation was conducted with 15 panelists to assess whether the control and experimental groups, following a 24‐h marination period and subsequent cooking, conformed to average consumer preferences. The sensory qualities of the samples were evaluated for taste, smell, aroma, flavor, color, tenderness, juiciness, and overall acceptability on a scale of 1 (inedible) to 9 (like extremely). A score above 5 points is considered to be accepted by consumers. The results of the sensory evaluation are shown in Figure 7 and Table S7.

There was a significant difference in taste, smell, flavor, juiciness, aroma, and overall acceptability (*p* < 0.001), color (*p* < 0.05) between the control and treatment groups. No significant difference was found for tenderness (*p* > 0.05). While the control group scored highest on acceptability, taste, odor, aroma, and flavor parameters, the 0.30% (v/v) eugenol marinade was noted to improve the juiciness and tenderness of chicken breast fillets. The panelists indicated that there was no significant discrepancy in the color parameter between the groups except the sample containing 0.30% linalool (ML3), and the results showed that there was no statistically significant difference (*p* > 0.05). The control and ML1 samples had the highest overall acceptability scores, while linalool was the least favorite with a score of 5.58, which stands for neither like nor dislike. On the other hand, samples containing eugenol (ML4 and ML5) had higher scores for all parameters than those containing linalool (ML2 and ML3). The lower scores given by the panelists for the use of linalool in chicken meat are thought to be due to the predominant floral aroma of linalool, which may not always complement the sensory profile of chicken meat. On the other hand, eugenol, responsible for the distinctive aroma of cloves, offers a spicy and warm note, harmonizing harmoniously with the flavor of poultry. For this reason, it was rated higher by the panelists as more suitable for use in poultry meat and was preferred to linalool as it offers a more balanced and attractive flavor. In a study conducted on the effect of linalool on the sensory characteristics of chicken breast in terms of odor, texture, color, and overall acceptability parameters, the authors stated that the addition of linalool to chicken meat at a ratio of 0.15%–0.30% gained consumer acceptability with a score that was higher than the control (He et al. 2023). These results showed that the effects of active essential oil components on the sensory profile of food can vary depending on consumer preferences and the strong aromas of these components can have notably different impacts on consumer perceptions.

## 4 Conclusion

The traditional marination process is a long‐established practice in the meat industry. However, incorporating essential active compounds into marinades is an approach that is gaining popularity. This innovative approach offers several potential advantages, such as improving shelf life and safety, providing a cheap, practical, environmentally friendly approach that reduces the need for artificial preservatives, and aligning with growing consumer demand for more natural, clean‐label products. Additionally, these compounds can enhance flavor and texture, offering a more sophisticated and tailored product. The present study demonstrated that, depending on the concentration used, the addition of linalool and eugenol to the marinade reduced the counts of TVC, *Pseudomonas* spp., LAB, and yeast‐mold to varying degrees compared to the control group over a 9‐day storage period. The marinade containing linalool exhibited significant antibacterial activity during the 9 days of storage, whereas the marinade with eugenol showed remarkable anti‐yeast activity. Therefore, this study offers a potential approach to the use of active essential oil components as natural and effective antimicrobial agents to extend the shelf life of poultry meat by controlling the growth of spoilage and pathogenic microorganisms. However, additional studies are necessary to ascertain the effectiveness of active essential oil components on lipid oxidation and the texture profile of poultry meat during storage.

## Author Contributions

**Merva Nur Atasoy:** formal analysis (equal), investigation (equal). **Bahar Tuba Findik:** conceptualization (equal), data curation (equal), formal analysis (equal), investigation (equal), methodology (equal), writing – review and editing (equal). **Hilal Yildiz:** conceptualization (equal), data curation (equal), formal analysis (equal), investigation (equal), methodology (equal), supervision (equal), writing – review and editing (equal).

## Conflicts of Interest

The authors declare no conflicts of interest.

## Supporting information


**Table S1:** pH values of control and marinated chicken breast fillets during storage period.
**Table S2:** The total count of aerob mesophilic bacteria of control and marinated chicken breast fillets stored under vacuum for 9 days at 4°C.
**Table S3:** The *Pseudomonas* spp. count of control and marinated chicken breast fillets stored under vacuum for 9 days at 4°C.
**Table S4:** The LAB count of control and marinated chicken breast fillets stored under vacuum for 9 days at 4°C.
**Table S5:** The total coliform bacteria count of control and marinated chicken breast fillets stored under vacuum for 9 days at 4°C.
**Table S6:** The mold and yeast count of control and marinated chicken breast fillets stored under vacuum for 9 days at 4°C.
**Table S7:** The hedonic sensory evaluation of control and marinated chicken breast fillets.

## Data Availability

All data generated or analyzed during this study are included in this published article. Data from Merva Nur Atasoy's master's thesis were used to prepare this article.
