# Supplementary material for: Effect of Plum‐Sour‐Based Marination Liquids Enriched With Linalool and Eugenol on the Microbiological and Sensory Quality of Chicken Breast Meat
Source: Food Sci Nutr. 2025 Jun 2;13(6):e70361. doi: 10.1002/fsn3.70361 (PMC12129824; doi:10.1002/fsn3.70361)
Supplement: Supplementary file 1 — Table S1: pH values of control and marinated chicken breast fillets during storage period. Table S2: The total count of aerob mesophilic bacteria of control and marinated chicken breast fillets stored under vacuum for 9 days at 4°C. Table S3: The Pseudomonas spp. count of control and marinated chicken breast fillets stored under vacuum for 9 days at 4°C. Table S4: The LAB count of control and marinated chicken breast fillets stored under vacuum for 9 days at 4°C. Table S5: The total coliform bacteria count of control and marinated chicken breast fillets stored under vacuum for 9 days at 4°C. Table S6: The mold and yeast count of control and marinated chicken breast fillets stored under vacuum for 9 days at 4°C. Table S7: The hedonic sensory evaluation of control and marinated chicken breast fillets. [file FSN3-13-e70361-s001.docx]

***Supplementary materials for***

**Effect of plum-sour-based marination liquids enriched with linalool and eugenol on the microbiological and sensory quality of chicken breast meat Merva Nur Atasoy^1^, Bahar Tuba Findik^2^, Hilal Yildiz^1*^**

^1^Nevsehir Hacı Bektas Veli University, Faculty of Engineering and Architecture, Department of Food Engineering, Nevsehir, Türkiye

*E-mail address*: [mervanurilikhan@gmail.com](mailto:mervanurilikhan@gmail.com)

ORCID: 0000-0002-3726-2595

^2^Nevsehir Hacı Bektas Veli University, Faculty of Arts and Sciences, Department of Chemistry, Nevsehir, Türkiye

*E-mail address:* [btfindik@nevsehir.edu.tr](mailto:btfindik@nevsehir.edu.tr)

ORCID: 0000-0002-0030-4106

^3*^Nevsehir Hacı Bektas Veli University, Faculty of Engineering and Architecture, Department of Food Engineering, Nevsehir, Türkiye

*Correspondence:

*E-mail address*: [hilalyildiz@nevsehir.edu.tr](mailto:hilalyildiz@nevsehir.edu.tr)

ORCID: 0000-0002-7966-455X

Table S1. pH values of control and marinated chicken breast fillets during storage period

| **pH**  **Storage period (day)** | | | | |
| --- | --- | --- | --- | --- |
| Samples | 0 | 3 | 6 | 9 |
| C | 6.40±0.03^aA^ | 6.22±0.06^aB^ | 6.21±0.05^aB^ | 6.20±0.03^aB^ |
| ML1 | 3.89±0.02^bB^ | 4.17±0.03^bA^ | 4.16±0.03^bA^ | 4.14±0.02^cA^ |
| ML2 | 3.90±0.07^bC^ | 4.11±0.01^cB^ | 4.17±0.03^bAB^ | 4.23±0.03^bA^ |
| ML3 | 3.90±0.03^bC^ | 4.02±0.02^dB^ | 4.05±0.04^cdAB^ | 4.08±0.01^dA^ |
| ML4 | 3.87±0.02^bC^ | 4.03±0.02^dB^ | 4.00±0.05^dBC^ | 4.23±0.02^bA^ |
| ML5 | 3.85±0.03^bD^ | 4.04±0.03^dB^ | 4.10±0.02^bcA^ | 3.96±0.03^eC^ |

Values are expressed as mean ± standard error.

^a-d^Within each column, mean values with different superscripts (lowercase letters) are significantly different (*p*<.000; *p* <.001) for each pH value at the same day of storage.

^A-E^Within each row, values with different superscripts (capital letters) are significantly different (*p* <.000) for different experimental groups of the same sample during the storage period.

C: Chicken breast fillets without any marination; ML1: Marination liquid [plum sour and distiled water (1:5 ratio)]; ML2: ML1 + Linalool (L; 0.15%; v/v); ML3: ML1 + Linalool (L; 0.30%; v/v); ML4: ML1 + Eugenol (E; 0.15%; v/v); ML5: ML1 + Eugenol (E; 0.30%; v/v).

Table S2. The total count of aerob mesophilic bacteria of control and marinated chicken breast fillets stored under vacuum for 9 days at 4 °C

| **TVC (log CFU/g)**  **Storage period (day)** | | | | |
| --- | --- | --- | --- | --- |
| Samples | 0 | 3 | 6 | 9 |
| C | 5.280±0.020^aC^ | 5.410±0.120 ^aBC^ | 5.610±0.130 ^aB^ | 5.850±0.130 ^aA^ |
| ML1 | 3.780±0.080^eD^ | 4.667±0.119 ^bC^ | 5.016±0.125 ^bB^ | 5.323±0.106 ^bA^ |
| ML2 | 3.493±0.085^cC^ | 3.586±0.015 ^deC^ | 3.833±0.085 ^dB^ | 4.090±0.090 ^dB^ |
| ML3 | 3.150±0.150^dC^ | 3.460±0.090 ^eB^ | 3.497±0.015 ^eAB^ | 3.667±0.060 ^eA^ |
| ML4 | 3.690±0.200^bD^ | 3.940±0.010 ^cC^ | 4.210±0.096 ^cB^ | 4.387±0.035 ^cA^ |
| ML5 | 3.503±0.015^cC^ | 3.633±0.040 ^dB^ | 3.900±0.075 ^dA^ | 3.957±0.060 ^dA^ |

Values are expressed as mean ± standard error.

^a-e^Within each column, mean values with different superscripts (lowercase letters) are significantly different (*p*<.000) for each TVC count at the same day of storage.

^A-D^Within each row, values with different superscripts (capital letters) are significantly different (*p* <.001) for different experimental groups of the same sample during the storage period.

C: Chicken breast fillets without any marination; ML1: Marination liquid [plum sour and distiled water (1:5 ratio)]; ML2: ML1 + Linalool (L; 0.15%; v/v); ML3: ML1 + Linalool (L; 0.30%; v/v); ML4: ML1 + Eugenol (E; 0.15%; v/v); ML5: ML1 + Eugenol (E; 0.30%; v/v).

Table S3. The *Pseudomonas* spp. count of control and marinated chicken breast fillets stored under vacuum for 9 days at 4 °C

| ***Pseudomonas* spp. (log CFU/g)**  **Storage period (day)** | | | | |
| --- | --- | --- | --- | --- |
| Samples | 0 | 3 | 6 | 9 |
| C | 5.470±0.097 ^aD^ | 5.776±0.025 ^aC^ | 6.530±0.082 ^aA^ | 6.090±0.182 ^aB^ |
| ML1 | 3.747±0.085 ^cD^ | 4.030±0.044 ^cC^ | 5.100±0.066 ^bB^ | 5.340±0.053 ^bA^ |
| ML2 | 3.467±0.223 ^dC^ | 4.026±0.055 ^cB^ | 4.137±0.127 ^cB^ | 4.783±0.161 ^cA^ |
| ML3 | 2.778±0.095 ^fC^ | 3.787±0.021 ^dB^ | 4.003±0.025 ^cA^ | 4.123±0.109 ^dA^ |
| ML4 | 4.720±0.036 ^bC^ | 4.750±0.046 ^bBC^ | 4.847±0.067 ^bB^ | 4.973±0.085 ^cA^ |
| ML5 | 3.043±0.093 ^eB^ | 3.890±0.295 ^cdA^ | 3.943±0.339 ^cA^ | 3.993±0.130 ^dA^ |

Values are expressed as mean ± standard error.

^a-f^Within each column, mean values with different superscripts (lowercase letters) are significantly different (*p*<.000) for each *Pseudomonas* spp. count at the same day of storage

^A-D^Within each row, values with different superscripts (capital letters) are significantly different (*p* <.000) for different experimental groups of the same sample during the storage period.

C: Chicken breast fillets without any marination; ML1: Marination liquid [plum sour and distiled water (1:5 ratio)]; ML2: ML1 + Linalool (L; 0.15%; v/v); ML3: ML1 + Linalool (L; 0.30%; v/v); ML4: ML1 + Eugenol (E; 0.15%; v/v); ML5: ML1 + Eugenol (E; 0.30%; v/v).

Table S4. The LAB count of control and marinated chicken breast fillets stored under vacuum for 9 days at 4 °C

| **LAB (log CFU/g)**  **Storage period (day)** | | | | |
| --- | --- | --- | --- | --- |
| Samples | 0 | 3 | 6 | 9 |
| C | 3.717±0.035 ^aA^ | 2.843±0.040 ^aC^ | 3.557±0.055 ^bB^ | 3.720±0.020 ^aA^ |
| ML1 | 3.697±0.016 ^aA^ | 2.417±0.065 ^bC^ | 3.767±0.068 ^aA^ | 2.750±0.091 ^bB^ |
| ML2 | 2.520±0.030 ^dB^ | 2.216±0.031 ^dD^ | 3.613±0.030 ^bA^ | 2.393±0.059 ^cC^ |
| ML3 | 2.370±0.060 ^eB^ | 2.020±0.027 ^eC^ | 3.560±0.056 ^bA^ | 2.040±0.040 ^aC^ |
| ML4 | 2.763±0.015 ^bB^ | 2.313±0.042 ^cD^ | 3.610±0.063 ^bA^ | 2.483±0.025 ^cC^ |
| ML5 | 2.663±0.030 ^cB^ | 2.010±0.010 ^eC^ | 2.853±0.055 ^cA^ | 2.287±0.070 ^dC^ |

Values are expressed as mean ± standard error.

^a-e^Within each column, mean values with different superscripts (lowercase letters) are significantly different (*p*<.000) for each LAB count at the same day of storage

^A-D^Within each row, values with different superscripts (capital letters) are significantly different (*p* <.000) for different experimental groups of the same sample during the storage period.

C: Chicken breast fillets without any marination; ML1: Marination liquid [plum sour and distiled water (1:5 ratio)]; ML2: ML1 + Linalool (L; 0.15%; v/v); ML3: ML1 + Linalool (L; 0.30%; v/v); ML4: ML1 + Eugenol (E; 0.15%; v/v); ML5: ML1 + Eugenol (E; 0.30%; v/v).

Table S5. The total coliform bacteria count of control and marinated chicken breast fillets stored under vacuum for 9 days at 4 °C

| **Total coliform (log CFU/g)**  **Storage period (day)** | | | | |
| --- | --- | --- | --- | --- |
| Samples | 0 | 3 | 6 | 9 |
| C | 2.820±0.026 ^aB^ | 2.877±0.032 ^aB^ | 2.923±0.025 ^aB^ | 3.447±0.127 ^aA^ |
| ML1 | < 10^1^ | < 10^1^ | < 10^1^ | < 10^1^ |
| ML2 | < 10^1^ | < 10^1^ | < 10^1^ | < 10^1^ |
| ML3 | < 10^1^ | < 10^1^ | < 10^1^ | < 10^1^ |
| ML4 | < 10^1^ | < 10^1^ | < 10^1^ | < 10^1^ |
| ML5 | < 10^1^ | < 10^1^ | < 10^1^ | < 10^1^ |

Values are expressed as mean ± standard error.

^a-c^Within each column, mean values with different superscripts (lowercase letters) are significantly different (*p*<.000) for each total colifom counts at the same day of storage

^A-B^Within each row, values with different superscripts (capital letters) are significantly different (*p* <.000) for different experimental groups of the same sample during the storage period.

C: Chicken breast fillets without any marination; ML1: Marination liquid [plum sour and distiled water (1:5 ratio)]; ML2: ML1 + Linalool (L; 0.15%; v/v); ML3: ML1 + Linalool (L; 0.30%; v/v); ML4: ML1 + Eugenol (E; 0.15%; v/v); ML5: ML1 + Eugenol (E; 0.30%; v/v).

Table S6. The mold and yeast count of control and marinated chicken breast fillets stored under vacuum for 9 days at 4 °C

| **Mold and yeast (log CFU/g)**  **Storage period (day)** | | | | |
| --- | --- | --- | --- | --- |
| Samples | 0 | 3 | 6 | 9 |
| C | 3.697±0.068 ^aC^ | 3.720±0.030 ^bC^ | 3.910±0.066 ^cB^ | 4.477±0.150 ^bcA^ |
| ML1 | 3.443±0.041 ^dD^ | 3.877±0.117 ^aC^ | 4.583±0.076 ^aB^ | 5.353±0.050 ^aA^ |
| ML2 | 3.427±0.021 ^deD^ | 3.940±0.040 ^aC^ | 4.223±0.100 ^bB^ | 4.703±0.195 ^bA^ |
| ML3 | 3.360±0.044 ^eD^ | 3.747±0.045 ^bC^ | 3.963±0.047 ^cB^ | 4.397±0.195 ^cA^ |
| ML4 | 3.602±0.027 ^cA^ | 3.530±0.101 ^cB^ | 3.480±0.026 ^dB^ | 2.710±0.010 ^dC^ |
| ML5 | 3.657±0.031 ^bA^ | 2.943±0.040 ^dA^ | 2.657±0.031 ^eB^ | 2.610±0.040 ^dB^ |

Values are expressed as mean ± standard error.

^a-e^Within each column, mean values with different superscripts (lowercase letters) are significantly different (*p*<.000) for each mold and yeast count at the same day of storage.

^A-D^Within each row, values with different superscripts (capital letters) are significantly different (*p* <.000) for different experimental groups of the same sample during the storage period.

C: Chicken breast fillets without any marination; ML1: Marination liquid [plum sour and distiled water (1:5 ratio)]; ML2: ML1 + Linalool (L; 0.15%; v/v); ML3: ML1 + Linalool (L; 0.30%; v/v); ML4: ML1 + Eugenol (E; 0.15%; v/v); ML5: ML1 + Eugenol (E; 0.30%; v/v).

TableS7. The hedonic sensory evaluation of control and marinated chicken breast fillets

| Samples | Taste | Smell | Aroma | Flavor | Color | Tenderness | Juiciness | Overall acceptability |
| --- | --- | --- | --- | --- | --- | --- | --- | --- |
| C | 6.87±1.69^a^ | 7.36±1.58^ca^ | 7.13±1.52^a^ | 6.73±1.25^a^ | 7.33±1.14^a^ | 7.00±1.37^ab^ | 6.73±1.13^bc^ | 7.20±0.84^a^ |
| ML1 | 6.18±1.45^bc^ | 6.91±0.83^ab^ | 6.24±1.11^b^ | 6.33±1.26^a^ | 7.07±1.07^ab^ | 6.87±0.89^b^ | 6.91±0.63^ab^ | 6.73±0.94^a^ |
| ML2 | 5.53±1.60^cd^ | 5.69±1.24^c^ | 6.29±1.63^b^ | 5.29±1.65^b^ | 6.82±1.25^ab^ | 6.91±1.12^ab^ | 6.33±1.33^c^ | 5.73±1.40^b^ |
| ML3 | 5.36±1.37^d^ | 5.78±1.13^c^ | 5.93±1.25^b^ | 5.47±1.31^b^ | 6.53±1.29^b^ | 7.00±1.22^ab^ | 6.98±1.03^ab^ | 5.58±1.27^b^ |
| ML4 | 6.27±1.54^ab^ | 6.38±1.32^b^ | 6.00±1.48^b^ | 5.51±1.46^b^ | 7.07±1.18^ab^ | 7.16±1.31^ab^ | 7.16±1.09^ab^ | 6.09±1.22^b^ |
| ML5 | 6.07±1.42^bc^ | 6.49±1.31^b^ | 6.07±1.38^b^ | 5.60±1.54^b^ | 6.82±1.00^ab^ | 7.44±1.18^a^ | 7.29±0.92^a^ | 6.42±1.44^ab^ |
| ***p*** | 0.000 | 0.000 | 0.001 | 0.000 | 0.029 | 0.220 | 0.000 | 0.000 |

Values are expressed as mean ± standard error.

^a-d^Within each column, mean values with different superscripts (lowercase letters) are significantly different (*p*<.000)

C: Chicken breast fillets without any marination; ML1: Marination liquid [plum sour and distiled water (1:5 ratio)]; ML2: ML1 + Linalool (L; 0.15%; v/v); ML3: ML1 + Linalool (L; 0.30%; v/v); ML4: ML1 + Eugenol (E; 0.15%; v/v); ML5: ML1 + Eugenol (E; 0.30%; v/v).
